# Effect of Ovocystatin on Amyloid β 1-42 Aggregation—In Vitro Studies

**DOI:** 10.3390/ijms24065433

**Published:** 2023-03-12

**Authors:** Bartłomiej Stańczykiewicz, Tomasz M. Goszczyński, Paweł Migdał, Marta Piksa, Krzysztof Pawlik, Jakub Gburek, Krzysztof Gołąb, Bogusława Konopska, Agnieszka Zabłocka

**Affiliations:** 1 Division of Consultation Psychiatry and Neuroscience, Department of Psychiatry, Wroclaw Medical University, L. Pasteura 10, 50-367 Wroclaw, Poland; 2 Department of Experimental Oncology, Hirszfeld Institute of Immunology and Experimental Therapy, Polish Academy of Sciences, R. Weigla 12, 53-114 Wroclaw, Poland; 3 Department of Microbiology, Hirszfeld Institute of Immunology and Experimental Therapy, Polish Academy of Sciences, Weigla 12, 53-114 Wroclaw, Poland; 4 Department of Pharmaceutical Biochemistry, Division of Pharmaceutical Biochemistry, Wroclaw Medical University, Borowska 211 A, 50-556 Wroclaw, Poland

**Keywords:** ovocystatin, amyloid beta peptide, ThT, CD, TEM, Alzheimer’s disease

## Abstract

Amyloid β peptides (Aβ) aggregating in the brain have a potential neurotoxic effect and are believed to be a major cause of Alzheimer’s disease (AD) development. Thus, inhibiting amyloid polypeptide aggregation seems to be a promising approach to the therapy and prevention of this neurodegenerative disease. The research presented here is directed at the determination of the inhibitory activity of ovocystatin, the cysteine protease inhibitor isolated from egg white, on Aβ42 fibril genesis in vitro. Thioflavin-T (ThT) assays, which determine the degree of aggregation of amyloid peptides based on fluorescence measurement, circular dichroism spectroscopy (CD), and transmission electron microscopy (TEM) have been used to assess the inhibition of amyloid fibril formation by ovocystatin. Amyloid beta 42 oligomer toxicity was measured using the MTT test. The results have shown that ovocystatin possesses Aβ42 anti-aggregation activity and inhibits Aβ42 oligomer toxicity in PC12 cells. The results of this work may help in the development of potential substances able to prevent or delay the process of beta-amyloid aggregation—one of the main reasons for Alzheimer’s disease.

## 1. Introduction

Neurodegeneration is a progressive process of neurons’ destruction that leads to their death, entailing, e.g., abnormalities in signal transduction pathways controlling neuronal functions. At the cellular level, neurodegenerative processes are promoted by oxidative stress [1,2,3], mitochondrial dysfunction [4], trophic factor deficiency [5,6], and excessive secretion of pro-inflammatory mediators [7]. In the development of neurodegenerative diseases, one of the main risk factors is aging, which, with genetic and environmental factors, leads to the manifestation of the illness. The scientific literature has presented many mechanisms contributing to Alzheimer’s disease (AD), including amyloid-β (Aβ), protein tau, and apolipoprotein E (APOE), as the crucial elements of the AD pathophysiology [8]. It has been extensively demonstrated that mutations in amyloid precursor protein (*APP*) and presenilin 1 (*PSEN1*) and 2 (*PSEN2*) genes lead to the development of AD and the production of toxic Aβ peptides [9,10]. Thus, the deposition of amyloid beta 1–42 (Aβ42) is one of the most important pathological factors in AD, resistant to the action of proteolytic enzymes, showing toxic effects by inducing inflammation and finally neuronal cell death [11]. It has been shown that soluble Aβ species, mainly Aβ42 oligomers, exert a pivotal role in the pathogenesis of synaptic damage, especially in the early stage of AD [12]. It has also been established that Aβ—induced neurotoxicity occurs mainly through the induction of apoptotic pathways in neurons [13,14].

Many studies have demonstrated that cysteine proteases constitute an important factor in neurogenesis as well as in the pathology of neurodegenerative disorders. Indeed, cysteine protease inhibitor—cystatin C (Cys C)—is implicated in neuroprotection and repair in the nervous system in response to diverse neurotoxic conditions [15]. It has been widely indicated that Cys C plays a pivotal biological role in Alzheimer’s disease [16]. Firstly, Cys C co-deposits with Aβ plaques in patients with AD brains. Additionally, a specific, saturable, and high-affinity binding between Cys C and both Aβ42 and Aβ40 has been determined [17]. Secondly, it has been reported that Cys C possesses inhibitory properties against amyloid fibril formation and oligomerization [18]. Additionally, Mi et al. suggested that endogenous Cys C is a carrier of soluble Aβ in the brain, blood, and cerebrospinal fluid (CSF), where it inhibits Aβ aggregation into insoluble plaques [19]. Finally, neuroprotective roles of Cys C, including the inhibition of cysteine proteases, such as cathepsins B, H, K, L, and S, the induction of autophagy, and regulation of cell proliferation, have been demonstrated [16,20].

Considering these findings, it becomes increasingly important to develop new therapeutic strategies, such as nutraceutical drugs, which are potentially neuroprotective. Indeed, bioactive peptides/proteins are promising candidates for use as the inhibitors of Aβ fibril formation, such as ovocystatin (ovoC). Interestingly, ovocystatin improves cognitive function in young rats [21] and prevents aging-related cognitive impairment in older animals [22]. Moreover, recently published studies showed that the administration of ovoC in drinking water might be effective in the prevention of cognitive deterioration in APP/PS1 mice [23]. Additionally, ovoC could also be applied as a useful factor against Aβ oligomerization and consequent amyloid fibril formation and tau protein deposition [24]. OvoC is a small protein inhibitor of cysteine proteinases and the best model protein under study representing the cystatin superfamily [25]. Similar to Cys C, ovoC inhibits the lysosomal cathepsins, such as cathepsin B, H, K, L, and S [26], and it has comparable biological properties [27]. Moreover, it is highly homologous to human Cys C (62% structural similarity). Nevertheless, there is a lack of indication of the crucial properties of ovoC and its mechanisms of action, related to the pathology of aging-associated neurodegeneration.

Based on our animal model research results, this study aimed to assess the effect and mechanism of ovoC as an inhibitor of Aβ42 fibrillation. Our findings provide an important insight into a new potential anti-neurodegenerative approach for an efficient inhibitor that can reduce the intensity of Aβ42 fibril formation and grounds for developing adjuvant treatment strategies based on nutraceuticals.

## 2. Results

### 2.1. Isolation and Purification of Ovocystatin

**Figure 1:**
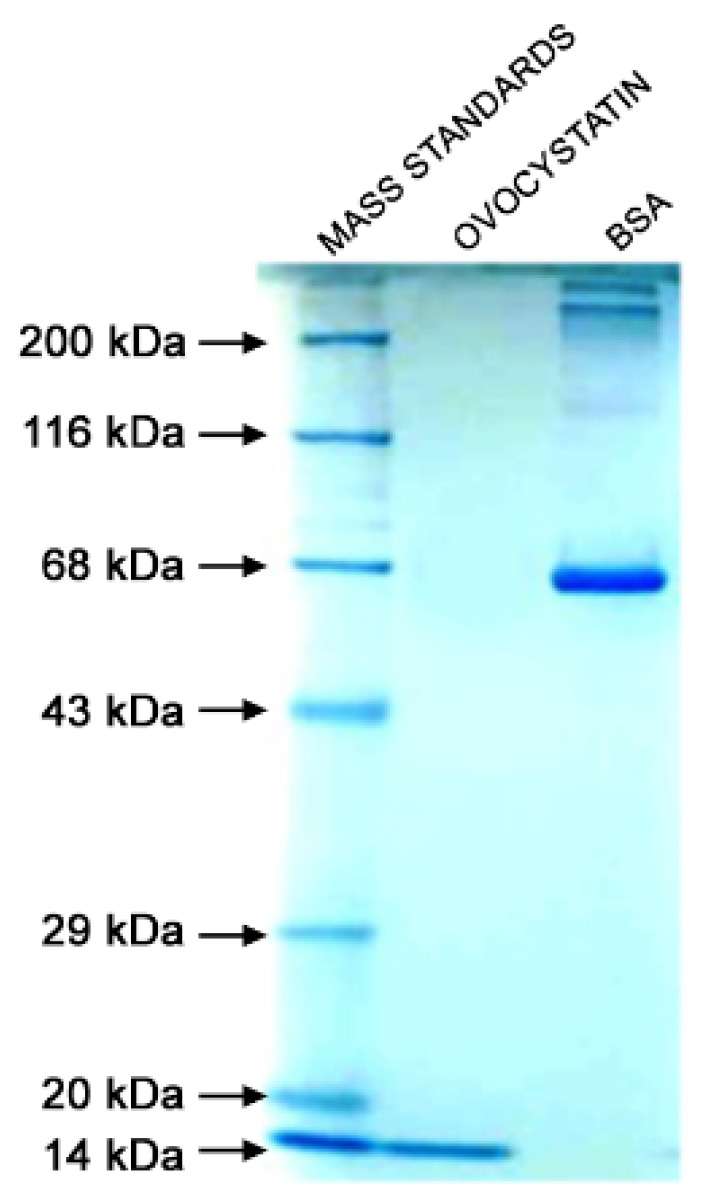
PAGE-SDS electrophoresis of preparation of ovocystatin in 10% gel (reducing conditions). The lanes were loaded according to the order (from left): 10 µL of the mass standards (Protein Marker III), 10 µL of 0.2 mg/mL ovocystatin, 10 µL of 0.2 mg/mL BSA, and separation was carried out in 25 mM TRIS/192 mM glycine buffer pH 8.3 with 0.1% SDS for 90 min. The bovine serum albumin was loaded as an additional control.

**Figure 2:**
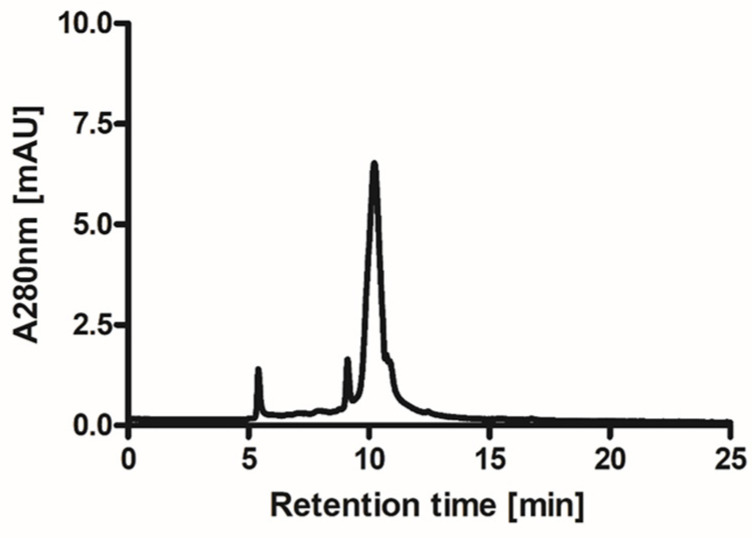
HPLC gel filtration is the final ovocystatin preparation. Twenty microliters of the sample were loaded on the Bio SEC-5 column and eluted with 150 mmol/L sodium phosphate, pH 7.4. Protein in the eluent was detected by UV absorption measurement at 280 nm. The retention time determined for ovocystatin was 10.2 min.

The preparation of ovoC from 20 eggs typically yielded 17–18 mg of the pure inhibitor with excellent biological activity ranging in U/mg between 23 and 25 as assessed using Barrett’s colorimetric method. The isolation and lyophilization conditions did not induce polymerization of ovoC and allowed us to obtain homogenous preparation of inhibitor. The isolation and lyophilization conditions did not induce polymerization of ovoC and allowed us to obtain homogenous preparation of inhibitor. The molecular weight of the obtained protein is almost the same migration distance of the 14 kDa molecular weight marker and agrees with the chicken egg white cystatin molecular weight of approximately 13 kDa (Figure 1). The purity of ovoC determined using HPLC was greater than 95% (Figure 2).

### 2.2. Effects of Ovocystatin against Aβ42 Fibrils Formation by ThT Assay

**Figure 3:**
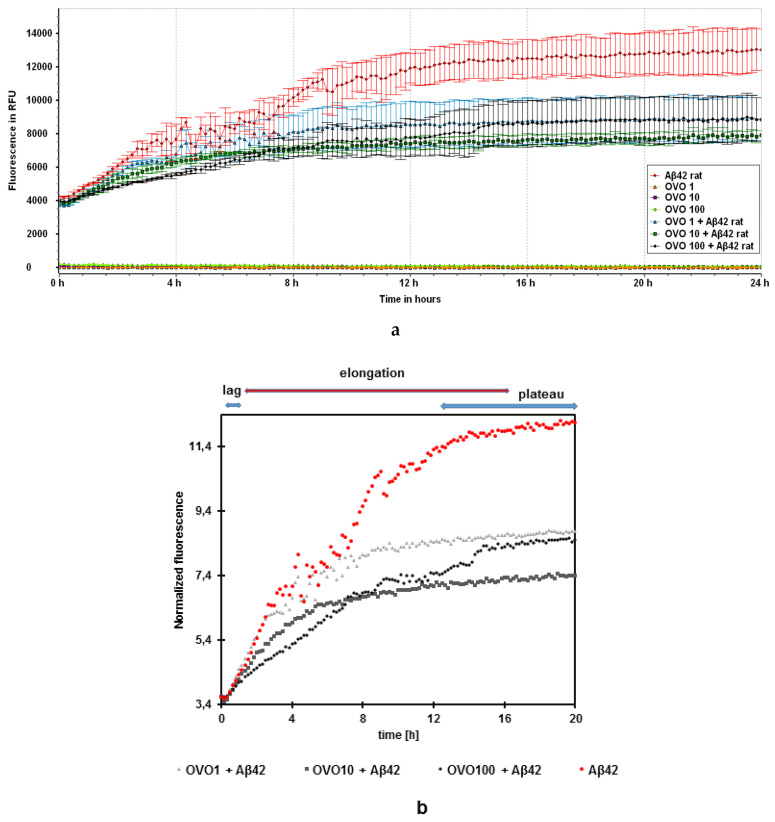

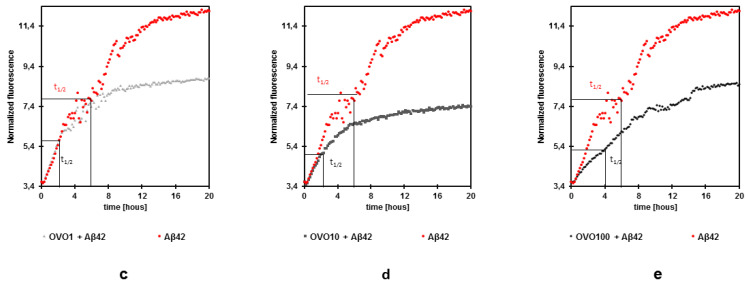
Efficacy of ovocystatin against Aβ42 aggregation. Aβ42 fibrils formation without or with ovocystatin (ovo) was monitored using ThT fluorescence assay. Fluorescence intensity was measured at an excitation wavelength of 420 nm and an emission wavelength of 480 nm (**a**). Normalized fluorescence curves with lag, elongation, and plateau phases (**b**), and half time of aggregation of Aβ42 (20 µM) alone, (**c**) Aβ42 with ovo 1 µg/mL, (**d**) Aβ42 with ovo 10 µg/mL, and (**e**) Aβ42 with ovo 100 µg/mL.

To explore if ovoC exerted an inhibitory effect on Aβ42 fibril formation, the ThT assay was employed (Figure 3). ThT selectively binds to the aggregated β-sheet fibrils of Aβ structures and leads to a significant increase in fluorescence of ThT proportional to the amount of the amyloid fibrils formed [28]. As can be seen in Figure 3a, under our experimental conditions (pH 7.4 and 37 °C), ovoC alone did not form fibrils; however, the Aβ42 solution presented a typical sigmoidal curve [29,30]. With the increasing incubation time, an augmentation in the number of fibrils was observed with the half-time (t_1/2_) of aggregation at 5.83 h (Figure 3c–e). The curve reached the plateau after 16 h, indicating the completion of amyloid fibril formation.

The kinetics of disaggregation of Aβ42 by ovoC are shown in Figure 3a (fluorescence in RFU) and Figure 3b (normalized fluorescence). When ovoC was incubated with Aβ42 solution, the fluorescence intensity at 480 nm decreased substantially, which indicated that the reduction in Aβ42 fibrillogenesis takes place. The changes are observed mainly in the elongation phase. Assuming that the Aβ42 plateau value is 100% of aggregation (RFU = 13,013), in the presence of ovoC, the level of amyloid fibrils was reduced to 68.7% for 1 µg/mL, 60.7% for 10 µg/mL, and 68.4% for 100 µg/mL of ovoC. The half-time of aggregation (t_1/2_) defined here as the time to reach half the maximum fluorescence intensity, varies depending on the ovoC concentration. With the increase in ovoC concentration, the time t_1/2_ increases, and the kinetics of aggregation changes (2.17 h for 1 µg/mL (Figure 3c), and 10 µg/mL (Figure 3d) of ovoC, and 4 h for 100 µg/mL of ovoC (Figure 3e)). The elongation curves for Aβ42 and ovoC (1 and 10 µg/mL) + Aβ42 almost overlap in the initial phase of elongation; however, the addition of ovoC causes a decrease in the possible level of saturation, and thus a decrease in the final fluorescence and shortening of the t_1/2_. However, the log phase of studied samples was also slightly lower when compared to Aβ42 alone, which may suggest the possibility of ovoC interaction with monomers, dimers, or oligomers. Thus, averaging the observations obtained from the three experiments, it is visible that ovocystatin reduces the aggregation of the Aβ42 peptides.

### 2.3. Effects of Ovocystatin on Aβ42 Fibril Morphology

**Figure 4:**
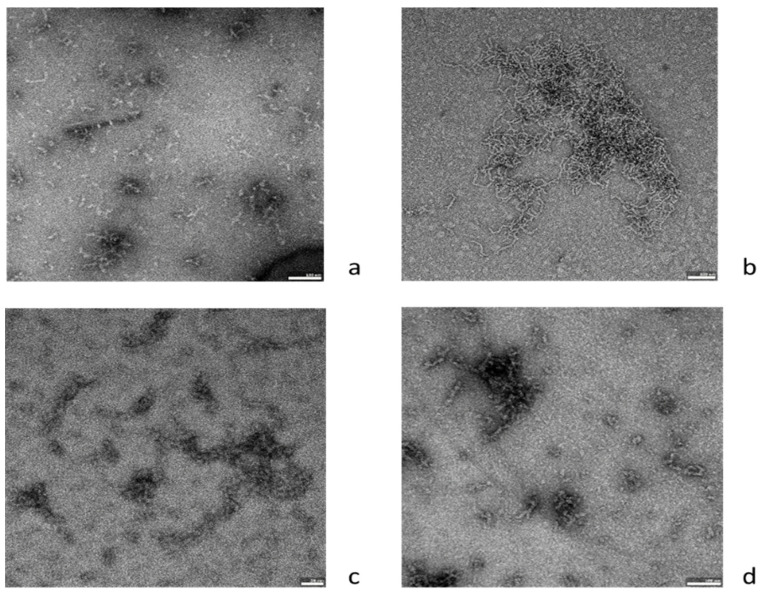
Representative transmission electron microscopy images of (**a**) Aβ42 at t = 0 (*n* = 43), (**b**) Aβ42 after 48 h incubation (*n* = 41), (**c**) Aβ42 + ovoC (10 μg/mL) after 48 h incubation (*n* = 48), and (**d**) Aβ42 + ovoC (100 μg/mL) after 48 h incubation (*n* = 39). Scale bars represent 0.5 μm.

To confirm the observed inhibitory effect of ovoC on Aβ42 aggregation, TEM was employed to visualize changes in fibril morphology. Aβ42 alone exhibited a large amount of long and thick amyloid fibrils creating large clusters after 48 h of incubation at 37 °C (Figure 4a,b). OvoC, which showed inhibitory activity in the ThT assay, significantly reduces Aβ42 fibril density and length, compared to the Aβ42 alone (Figure 4c,d). The highest concentration of ovoC: 100 µg/mL reduced the amounts of amyloid aggregates most effectively (Figure 4d). These results support the effects of *ovocystatin* against Aβ42 fibril formation by ThT assay.

### 2.4. Effects of Ovocystatin on Aβ42 Secondary Structure during Fibrillation

**Figure 5:**
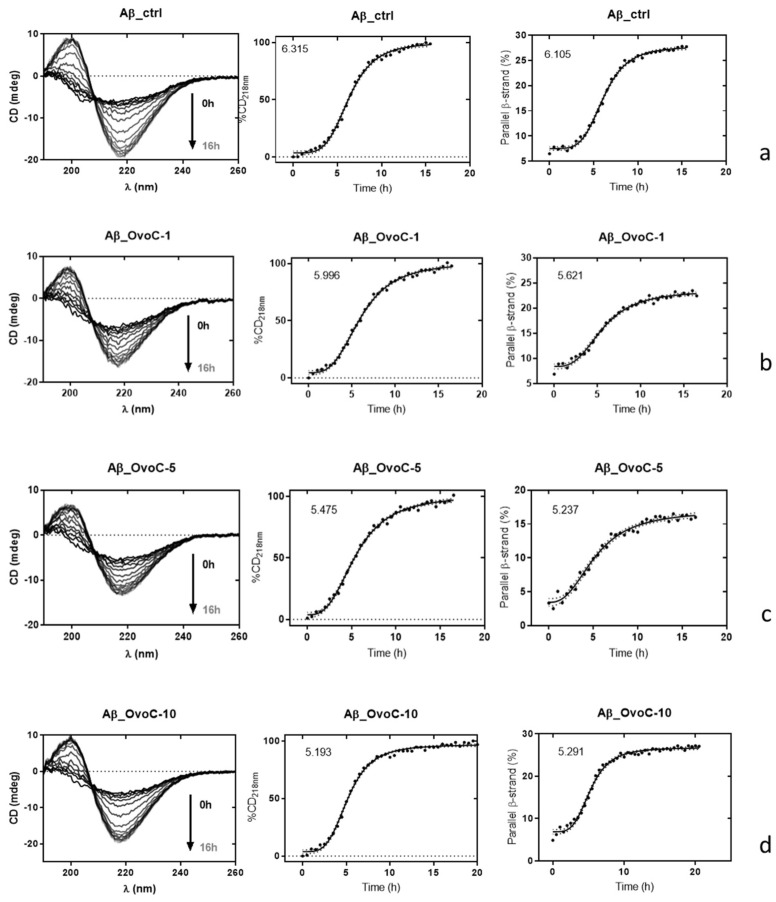
Circular dichroism spectra of amyloid β42 (Aβ) in the absence (**a**) and in the presence of ovocystatin at 1 μg/mL (**b**), 5 μg/mL (**c**), and 10 μg/mL (**d**) concentration. Ctrl—control; amyloid β alone—Aβ; ovoC—ovocystatin; amyloid β42 + ovocystatin: Aβ + ovoC.

CD spectroscopy in the far UV region (190–240 nm) was used to estimate changes in the secondary structure of the Aβ42 sample alone or incubated in the presence of ovoC. Using CD spectroscopy, the typical three phases process is measured, including a lag phase without significant conformation changes, an exponential phase including a rapid increase in β-sheet presence, and a plateau phase, in which the secondary structure of the β sheet dominates. The overlays of CD spectra of Aβ42 alone and Aβ42 + ovoC at concentrations of 1, 5, and 10 μg/mL are presented in Figure 5. As can be seen in Figure 5a, we observed the transition from the non-amyloidogenic unordered/α-helix of soluble Aβ42 to the amyloidogenic β-sheet conformation. After 5 h incubation, both Aβ42 peptide and Aβ42/ovoC mixture exhibited a strong positive band at 218 nm, suggesting the conformational changes of Aβ42 from α-helix to β-sheet state (IC_50_: 6.105, 5.621, 5.237 and 5.291 for Aβ42 alone, Aβ42 + ovoC 1 μg/mL, Aβ42 + ovoC 5 μg/mL and Aβ42+ovoC10 μg/mL, respectively). β-sheet secondary structure formation after 16 h is indicated by the appearance of a negative band at 218 nm and a positive band at 200 nm. For the Aβ42 sample treated with ovoC, no differences in the structural transition of Aβ42 were observed (Figure 5b–d) compared to those observed in the Aβ42 incubated alone (Figure 5a). These observations suggested that ovoC did not affect the intensity of the Aβ42 aggregates’ transformation from the initial structure to the β-sheet protein.

### 2.5. Ovocystatin Increased PC12 Cells Viability

**Figure 6:**
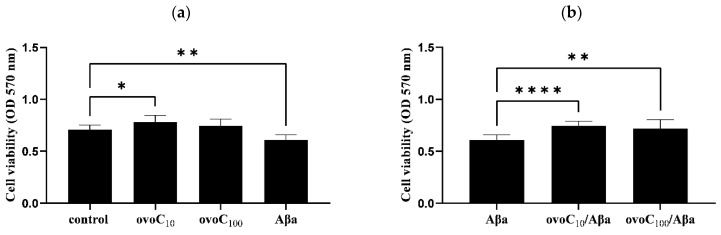
Viability of PC12 cells treated with Aβ1−40 (20 μM) alone (**a**) or with different concentrations of ovoC (**b**) for 24 h. Cell viability was measured with MTT assays and is shown as a percentage of the untreated cells (control). Data were analyzed by one-way ANOVA to evaluate treatment effect. * *p* ≤ 0.038 and ** *p* ≤ 0.002 when compared with control cells. **** *p* ≤ 0.0001 and ** *p* ≤ 0.01 when compared with Aβ42 alone.

To determine if ovoC (10 and 100 µg/mL) and Aβ42 affect PC12 cell viability, an MTT assay was performed. One-way ANOVA analysis revealed a 10% (OD_570_ = 0.783) increase in PC12 cell proliferation of ovoC (10 µg/mL)-treated PC12 cells, while ovoC (100 µg/mL) showed an effect comparable to the control cells (OD_570_ = 0.708) but is not toxic to cells (Figure 6a). Aβ42 reduced PC12 cell viability to 85% (OD_570_ = 0.608). To determine whether ovoC can inhibit Aβ42-dependent toxicity, Aβ42 (10µM) with and without ovoC (10 and 100 µg/mL) was added to PC12 cells for 24 h. An increase in PC12 cell viability was observed when Aβ42 was applied together with ovoC. Cell survival increased to 105% for ovoC (10 µg/mL)(OD_570_ = 0.743) and to 101% for ovoC (100 µg/mL)(OD_570_ = 0.717) compared to cells treated with Aβ42 alone (Figure 6b). Thus, this data indicate that ovocystatin increased the viability of Aβ42-treated cells.

## 3. Discussion

Alzheimer’s disease is the most frequent cause of dementia in the elderly. Unfortunately, there is no effective treatment able to prevent or stop AD. Up until now, clinical trials have focused mainly on patients who have developed symptoms. However, the importance of using therapies that could prevent the development of pathological changes in the brain has presently been highlighted [31]. The accumulation of insoluble amyloid β deposits forming the senile plaques is observed in the brain and is a hallmark of AD. Fibrillation proceeds via oligomers to protofibrils and fibrils of Aβ42 leading finally to the production of the insoluble and toxic senile plaques. Aggregated Aβ mainly takes the form of β-sheet conformation. This form seems to be extremally toxic to the neurons [32].

Biologically active substances, safe, bioavailable, and exhibiting neuroprotective potential may constitute an important therapeutic aspect in preventing and/or inhibiting the development of neurodegenerative diseases. One of them can be cystatin isolated from chicken egg white, named ovocystatin, which could be widely used in medical research due to its structural and biological similarities to human cystatin C possessing a beneficial effect on the inhibition of cysteine proteases and Aβ aggregation [19,33]. The ovocystatin preparation method developed by us allows us to obtain a pure ovocystatin preparation, without additional ingredients and impurities. The buffers containing volatile salts were used, and were removed during the lyophilization process. This preparation procedure makes it possible to obtain a monomeric form of ovocystatin with very high activity and purity reaching nearly 100%. Moreover, the inhibitor isolated by this method has already been tested in some studies, where its purity and cytotoxicity were also checked [34,35]. The results obtained indicate that our ovocystatin preparation is not contaminated with any substances.

It has been shown up until now, that ovocystatin has a beneficial impact on cognitive function in young rats, and might prevent aging-related cognitive impairment in older animals [21] and reduce memory decline in APP/PS1 mice model [23,24]. However, its potential molecular mechanism of action is not explained and needs to be examined in detail. It was determined by us in our latest paper that ovoC induced changes in the expression of Alzheimer’s disease—Aβ and tau proteins in APP/PS1 mice model [24]. Therefore, in this study, we focused our attention on examining the ability of ovoC to interact with Aβ42, inhibit fibrils, or control oligomer growth into non-toxic species. To follow the amyloid fibrils’ growth ThT fluorescence assay, TEM and CD were used. In ThT assay growth of fluorescence is the result of ThT binding to any β-sheet of amyloid, therefore studying this phenomenon does not provide information on the length of the resulting fibers but only on the total number of fibrillar structures [32,36]. Figure 3 shows, that after 24 h of incubation of Aβ42 at 37 °C, aggregation occurred in the form of a sigmoidal curve, in agreement with previous studies [29,30,37,38]. OvoC, at 1–100 µg/mL concentration could reduce amyloid fibril growth, picking at 10 µg/mL (39.3% inhibition). A total of 1 μg/mL and 100 μg/mL of ovoC reduced fibril formation up to 68.2%, and 68.4%, respectively, compared to Aβ alone taken as 100%. Additionally, the time t_1/2_ increases, and the kinetics of aggregation change with ovoC concentration (Figure 3c–e). The elongation curves for Aβ42 and ovoC (1 and 10 µg/mL) + Aβ42 almost overlap in the initial phase of elongation, however, the addition of ovoC causes a decrease in the possible level of saturation, and thus, a decrease in the final fluorescence and shortening of the t_1/2._ These observations indicate that ovoC may have anti-aggregative activity (especially at lower doses), contributing to PC12 increased viability, as was noticeable in the MTT assay (Figure 6). Similar activity was observed in other preparations, e.g., PRP complex [39,40], ginseng [41], baicalein [42], or tucaresol [43].

To provide more insight into the anti-fibrillation activity of ovoC, TEM observation was conducted to evaluate fibrillary morphology. As was shown, after 24 h of incubation, abundant amorphous and disordered aggregates were presented in the Aβ42 sample, and the aggregation process intensified significantly after 48 h of incubation (Figure 4a,b). However, TEM images of Aβ42 incubated with ovocystatin indicated the reduction in the Aβ fibril density and length (Figure 4c,d), compared with Aβ alone (Figure 4b). This means that ovoC can inhibit Aβ fibrillation. The images obtained by TEM confirmed our observation in the ThT fluorescence test.

Furthermore, CD spectroscopy was performed to explore the impact of ovocystatin on the Aβ42 peptide secondary structure. Figure 5 presents the changes in CD signals along with the varying amounts of ovoC added to Aβ42. Unfortunately, the results presented indicate that ovoC did not change the β-sheet secondary structure of Aβ42 with the lapse of time, which precludes ovocystatin potential action as a breaker of the amyloid β structure. Thus, the inhibition of amyloid aggregation observed in ThT may occur without affecting the changes in the β-sheet secondary structure of Aβ42. The ThT, TEM, and CD analysis data led us to propose the potential mechanism of ovoC action. Because changes were mainly observed in elongation phase, and slightly in the Aβ42 log phase (nucleation phase), the possibility of ovoC interaction with monomeric Aβ leading to inhibited seeding process is considered. However, it is more plausible that ovoC can stabilize the oligomers and/or protofibrils and slow down their conversion to fibrils. Such activity was also observed for human cystatin C [33,44] and some polyphenols, including, among others, resveratrol [45,46]. It was also demonstrated in the literature, that Aβ fibril formation can be controlled by specific amino acids within the Aβ peptide itself, and various (still not fully known and intensively studied) Aβ peptide regions contribute differently to Aβ aggregation and have identified crucial interactions among specific peptide regions controlling this process [47]. So, we hypothesize, that another possibility is to target, with ovoC, a specific subregion of Aβ42, blocking by this way the interactions between monomers and oligomers, thereby preventing the formation of further fibrils and aggregates.

OvoC was built up of a five-stranded anti-parallel β-sheet wrapping around a central α-helix. The linkage within the β-sheet is (N)-β1-(a)-β2-L1-β3-(AS)-β4-L2-β5-(C). As is a broad ‘appending structure’ located beyond the compact core of the molecule. It is probable that the interaction between ovoC and Aβ takes place via the C-terminal hydrophobic sequence (sequence: VYSIPWLNQIKLLESKC; L2-β5; aa 99–115). A similar interaction was reported for human cystatin C (sequence: IYAVPWQGTMTLSKS; L2-β5; aa 101–117). This region showed the highest inhibitory effect toward Aβ-fibril formation. The association of human cystatin C and Aβ was specific, saturable, and of high affinity. This region is located in the C-terminal part within the L2 loop and β5 strand of the protein that are exposed to the environment. The C-terminal epitope mediates the interaction of Aβ with the L2-β5 part without any restriction [48,49]. Human cystatin C is highly homologous to chicken ovoC (62% structural similarity) and shares similar biological properties [26]. Therefore, the results from studies on human cystatin C might be strongly supportive of interpreting the molecular mechanisms of ovoC interactions with other proteins [27]. In order to confirm the above hypotheses, it is necessary to conduct further studies with the use of selective negative controls, for example, low molecular weight compounds that mask this site or missense mutants that lack this region.

Numerous literature reports of experimental studies have focused on the effect of naturally occurring substances on self-aggregated Aβ peptides. In particular, products with minimal side effects with the potential ability to pass the blood–brain barrier (BBB) are of particular interest [45,46,50]. Alghazwi et al., [51] determined the inhibitory effects of *Ecklonia radiata* fractions onAβ42 amyloid fibrillation. It was also shown that polyphenolic-rich fucoidan samples isolated from *Fucus vesiculosus* possess high anti-aggregative activity [52]. Moreover, curcumin, one of the most common polyphenolic compounds, possesses comparable properties [53]. Molecular docking and dynamic studies demonstrated that natural compounds could bind Aβ25-35 and could break the peptide leading to losing a significant quantum of β-sheet content resulting in inhibiting Aβ25-35 aggregation [54].

The results obtained from the ThT assay, TEM, and CD suggested that ovocystatin inhibits the elongation phase of Aβ42 aggregation breaking Aβ42 fibrillation, which points to a more relevant role in the amyloid morphology changes. This study highlights the potential of ovocystatin as a promising neuroprotective compound that has anti-aggregation effects on Aβ42 formation. In the next studies, we will try to explain the inhibitory and disaggregation mechanisms of ovocystatin more specifically. Furthermore, it is crucial to examine its cytoprotective activity using cell lines as in vitro models and to study the preventive effect on cognitive function using transgenic animals as an in vivo experiment.

## 4. Materials and Methods

### 4.1. Materials

1,1,1,3,3,3-Hexafluoro-2-propanol (99%) (HFIP), Thioflavin T (ThT), papain from papaya latex, *N*_α_-Benzoyl-DL-arginine β-naphthylamide hydrochloride (BANA), Tris(hydroxymethyl)aminomethane (TRIS); glycine; N,N,N′,N′-Tetramethyl ethylenediamine (TEMED), sodium dodecyl sulfate (SDS), and bovine serum albumin (BSA) were purchased from Sigma (Saint Louis, MO, USA). Amyloid β42 (Aβ42) was obtained from Tocris (Bristol, UK). Protein Marker III was purchased from AppliChem (Darmstadt, Germany). SimplyBlue Safe Stain was purchased from Thermo Fisher Scientific (Waltham, MA, USA).

### 4.2. Ovocystatin Isolation

Ovocystatin was prepared from chicken egg white according to a method based on affinity chromatography with immobilized S-carboxymethylated papain as described by Anastasi et al. [55] with slight modifications [56] and dialyzed overnight against 50 mM ammonium bicarbonate with several changes of the dialysis buffer. The volume of the preparation and the protein concentration was measured to calculate the content of the inhibitor. The protein concentration was estimated using an extinction coefficient at 280 nm equal to 0.871 for 0.1% ovocystatin solution. The final preparation was aliquoted and lyophilized for long-term storage. The quality of the inhibitor was assessed concerning the antipapain activity, against N_α_-Benzoyl-DL-arginine β-naphthylamide (BANA) [57], and the electrophoretic distribution in SDS-PAGE using 10% resolving gel in reducing conditions [58]. The proteins were visualized with colloidal Coomassie SimplyBlue SafeStain. Gel filtration chromatography on an HPLC system (Agilent 1100, Agilent Technologies) was used for the determination of ovocystatin purity. Twenty microliters of the sample were loaded on a Bio SEC-5 column (300.0 × 7.8 mm secured with a Bio SEC-5 guard column (50.0 × 7.8 mm) and eluted with 150 mmol/L sodium phosphate, pH 7.4 at the flow rate 1.2 mL/min. The protein in the eluent was detected by UV absorption measurement at 280 nm.

### 4.3. Amyloid β42 Preparation for Thioflavin T (ThT) Assay and Transmission Electron Microscopy (TEM)

Aβ42 lyophilized powder was monomerized by a 20 min pretreatment with 1,1,1,3,3,3-hexafluoro-propanol (HFIP) at RT. The solution was then sonicated for 15 min, and finally, the solution was evaporated overnight at RT. The thin film obtained was dissolved in 0.1% NH_4_OH/H_2_O and sonicated for 5 min in an ice bath. Finally, the 250 µM stock solution obtained was immediately diluted to the required concentration for an experiment.

### 4.4. ThT Assay of Aβ42 Fibril Formation

To determine the amyloidogenic structure of Aβ42 Thioflavin T (ThT) assay was performed. This is a dye that exhibits a strong increase in its fluorescence during binding to the β-sheet structure of Aβ, thus enabling the quantification of the presence of fibrous species. ThT was dissolved in a sterile phosphate buffer (100 mM, pH 7.4) to a final concentration of 10 μM and vortex 10 min at RT. Aβ42 (10 μM) fibrillation was measured with or without the presence of ovoC studied at concentrations of 10, and 100 μg/mL, using 96-well black plates. ThT was added to the reaction mixture just before starting the measurement. The final volume of the samples was 100 μL. Kinetics fluorescent data were collected at 37 °C in triplicate using CLARIOstar^®^ Microplate Reader (BMG LabTech, Offenburg, Germany), with measurements acquired at 15 min intervals. The excitation and emission wavelengths were set at 440 and 480 nm, respectively. The experiment was repeated twice in three independent repetitions (*n* = 6). Pure ThT solution was used as a blank to overcome the autofluorescence issue. The single measurement point presented on the plot is based on an average value of three independent repetitions, and the error bars represent their standard deviation.

### 4.5. TEM

Samples: Aβ42 alone (10 μM), Aβ42 (10 μM) + ovoC (10 μg/mL), or Aβ42 (10 μM) + ovoC (100 μg/mL) were incubated for 0, 24, and 48 h at 37 °C. The final volume of the samples was 100 μL. The samples were centrifuged (5 min, 50 µf), and the obtained pellets were fixed in 2.5% glutaraldehyde (POCH) for 24 h. Next, the samples were centrifuged again (5 min, 50 µf). A total of 10 µL of the sample was placed on copper grids (400 Mesh) with formvar film and carbon coating (Agar Scientific, Stansted, UK). Prepared samples were contrasted and performed with 2% uranyl acetate (MicroShop, Piaseczno, Poland). Imaging was performed using a JEOL 1200 microscope (Peabody, MA, USA) JEOL Japan microscope.

### 4.6. Circular Dichroism Spectroscopy (CD)

#### 4.6.1. Aβ42 Preparation for CD Measurement

Aβ42 lyophilized powder was monomerized by a 20 min pretreatment with 1,1,1,3,3,3-hexafluoro-propanol (HFIP). The solution was then sonicated in an ice bath for 15 min, and finally, the solvent was evaporated overnight at RT. The resulting thin film was dissolved in NaOH (10 mM). A total of 20µL aliquot containing 41.7 µg Aβ42 was diluted using 180 µL phosphate buffer (10 mM) that contained NaCl (10 mM), pH 7.4. The final solution for CD measurement contained 200 µL of Aβ42 at a concentration of 47.1 µM at pH 7.6.

#### 4.6.2. CD

Samples: Aβ42 alone, Aβ42 + ovoC (1, 5, and 10 µg/mL), were analyzed. Spectra were recorded on a J-1500 spectropolarimeter (Jasco, Japan) equipped with a thermostated cell holder and a PM-539 detector. CD spectra were recorded in the spectral range 190–260 nm using a 0.1 cm path length quartz cell at 37 °C. Spectra were accumulated six times. All values were corrected for solvent contributions—phosphate buffer (10 mM) that contained NaCl (10 mM), pH 7.6. Measurement conditions: data pitch, bandwidth, and D.I.T. were 0.5 nm, 1 nm, and 1 s, respectively, at 50 nm min^−1^. In co-incubation experiments of Aβ42, the ovoC spectrum was subtracted from the mixture spectrum to obtain only Aβ42 CD contribution.

To follow conformational changes and for inhibition studies, the CD signal at 218 nm was plotted vs. the time of incubation. Additionally, we used the BeStSel web server, [59] which provides a method to analyze the CD spectra with detailed secondary structure information, including parallel and antiparallel sheets. Using the obtained data parallel, β-strand content (%) was plotted vs. time of incubation. Data analysis was performed using the GraphPad Prism 7.01 software (GraphPad Software Inc., San Diego, CA, USA).

### 4.7. Cell Viability Determination

#### 4.7.1. Cell Culture

PC12 (Tet-On) cell line (ClonTech), a rat pheochromocytoma cell line was maintained in Dulbecco’s Modified Eagle’s Medium (DMEM) with 10% fetal bovine serum (FBS), 5% horse serum, and penicillin-streptomycin (PS) at 37 °C, 5% CO_2_ in a humidified incubator with the culture medium changed once every three days.

#### 4.7.2. Amyloid Preparation for Cell Treatment

Aβ42 lyophilized powder was monomerized by a 20 min pretreatment with 1,1,1,3,3,3-hexafluoro-propanol (HFIP) at RT. The solution was then sonicated for 15 min, and finally, the solution was evaporated overnight at RT. The thin film obtained was dissolved in DMSO. Finally, the 250 µM stock solution obtained was immediately diluted in PBS to the required concentration for an experiment. To obtain oligomers Aβ42 was incubated for 24 h at 37 °C, 5% CO_2_ in a humidified incubator.

#### 4.7.3. MTT Reduction Assay

Cell viability was determined by colorimetric MTT assay [60,61]. Cells were seeded onto a flat-bottomed 96-well culture plate (1 × 10^4^ cells/well) and incubated for 24 h at 37 °C with ovoC preparation (10 and 100 µg/mL) applied to the cells in the presence or absence of 24 h—aggregated Aβ42 (10 µM). After cell treatment, an MTT solution (5 mg/mL) was added, and the cells were incubated again for 4 h to develop formazan crystals. The formazan crystals were solubilized by adding 100 μL DMSO and vigorously shaken to complete resolution. The absorbance was measured at 570 nm by an EnSpire™ 2300 microplate reader (Parkin Elmer, MA, USA).

### 4.8. Data Analysis and Graphical Visualization

Statistics and graphs were prepared using GraphPad Prism Software v9. Data are presented as mean ± SD. Data were analyzed using the Ordinary one-way ANOVA test. A value of *p* < 0.05 or less is considered statistically significant.

## 5. Conclusions

The aggregation process of Aβ42 monomers to potentially neurotoxic oligomers and fibrils seems responsible for the beginning of AD. The first stage of this process is connected with conformational changes to a β-sheet structure. Previous studies have shown that ovocystatin improves cognitive function in young rats, prevents aging-related cognitive impairment in older animals, and induces changes in the expression of Alzheimer’s disease—Aβ and tau proteins in the APP/PS1 mice model. In the present work, we presented the potential mechanism of action of ovocystatin isolated from egg white in vitro, on Aβ42 fibrillogenesis using ThT fluorescence assay, transmission electron microscopy, and circular dichroism spectroscopy and tested cell viability by MTT assay. Our study has demonstrated that ovocystatin may reduce amyloid fibril formation and Aβ42 aggregation, unfortunately without the ability to β-structure formation/destabilization. However, it was shown that ovocystatin reduced Aβ42 toxicity in PC12 cells. Further studies will focus on explaining the potential mechanism of Aβ42–ovocystatin interaction. It is also necessary to investigate whether the observed phenomenon will have a beneficial effect on the survival and functions of neurons. We hope that the results of this work may help in the development of an effective inhibitor able to prevent or delay the process of beta-amyloid aggregation.

## Data Availability

The datasets generated during the current study are available from the corresponding author on a reasonable request.
